# Hyperacute Excitotoxic Mechanisms and Synaptic Dysfunction Involved in Traumatic Brain Injury

**DOI:** 10.3389/fnmol.2022.831825

**Published:** 2022-02-24

**Authors:** Brendan Hoffe, Matthew R. Holahan

**Affiliations:** Department of Neuroscience, Carleton University, Ottawa, ON, Canada

**Keywords:** traumatic brain injury, synaptic dysfunction, excitotoxicity, dendritic spine, neurodegeneration

## Abstract

The biological response of brain tissue to biomechanical strain are of fundamental importance in understanding sequela of a brain injury. The time after impact can be broken into four main phases: hyperacute, acute, subacute and chronic. It is crucial to understand the hyperacute neural outcomes from the biomechanical responses that produce traumatic brain injury (TBI) as these often result in the brain becoming sensitized and vulnerable to subsequent TBIs. While the precise physical mechanisms responsible for TBI are still a matter of debate, strain-induced shearing and stretching of neural elements are considered a primary factor in pathology; however, the injury-strain thresholds as well as the earliest onset of identifiable pathologies remain unclear. Dendritic spines are sites along the dendrite where the communication between neurons occurs. These spines are dynamic in their morphology, constantly changing between stubby, thin, filopodia and mushroom depending on the environment and signaling that takes place. Dendritic spines have been shown to react to the excitotoxic conditions that take place after an impact has occurred, with a shift to the excitatory, mushroom phenotype. Glutamate released into the synaptic cleft binds to NMDA and AMPA receptors leading to increased Ca^2+^ entry resulting in an excitotoxic cascade. If not properly cleared, elevated levels of glutamate within the synaptic cleft will have detrimental consequences on cellular signaling and survival of the pre- and post-synaptic elements. This review will focus on the synaptic changes during the hyperacute phase that occur after a TBI. With repetitive head trauma being linked to devastating medium – and long-term maladaptive neurobehavioral outcomes, including chronic traumatic encephalopathy (CTE), understanding the hyperacute cellular mechanisms can help understand the course of the pathology and the development of effective therapeutics.

## Introduction

The synapse is regarded as the fundamental site of communication between cells of the nervous system and synaptic dysfunction may represent a choke point for the multitude of upstream factors and downstream responses that lead to neuron atrophy or death. Synaptic malleability is a key component to behavior and cognition, with the formation, reshaping and strengthening of synapses being a key feature underlying learning and memory (Bourne and Harris, 2007; González-Tapia et al., 2016; Noye Tuplin and Holahan, 2019). Prolonged pathological changes at the synaptic level have been shown to play a role in several psychiatric and neurodegenerative diseases, such as Alzheimer’s disease, Huntington’s disease, Fragile X syndrome and schizophrenia (Calabrese et al., 2006; Herms and Dorostkar, 2016).

Over the past 15 years there has been an increase in attention to the relationship between traumatic brain injury (TBI), Alzheimer’s disease and chronic traumatic encephalopathy (Omalu et al., 2005; McKee et al., 2013; Goldstein et al., 2014; Albayram et al., 2020). Given that TBI can be regarded as a singular event (Wojnarowicz et al., 2017), the cellular processes that take place at the synapse dictate how the cell responds to the change in environment. These cellular processes can be categorized into four main time phases: hyperacute, acute, subacute, and chronic (Guerriero et al., 2015; MacFarlane and Glenn, 2015). While much research exists on the changes occurring days to months after TBI, there is comparatively little research investigating the hyperacute synaptic changes occurring within minutes to hours. These short-term changes could reveal the pathological mechanisms that are responsible for the later stages of neurodegeneration which may help shed light on potential therapeutic routes for neurodegenerative diseases associated with head trauma. This review will focus on and highlight the synaptic dysfunction during the hyperacute phase post-TBI, combining crucial findings from both *in vitro* and *in vivo* data to better understand what occurs at the synapse within minutes to hours after an impact has occurred.

## Time Phase Following Impact

**FIGURE 1:**
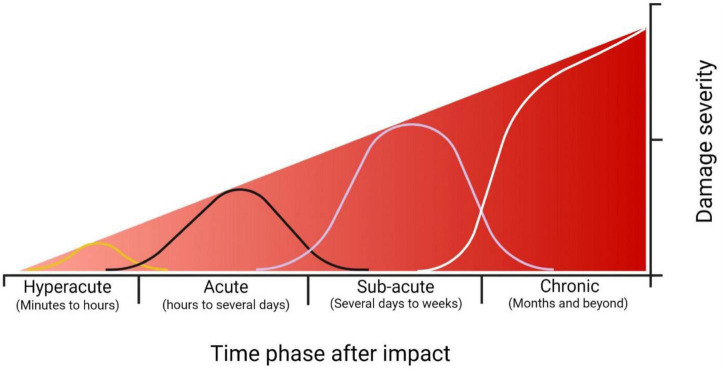
Timescale representation of the severity of damage following impact. Each time phase has the capacity to re-establish homeostasis through cellular repair or some form of treatment. If the cellular homeostasis is unresolved, however, the damage severity increases as time progresses. At the hyperacute time phase (minutes to hours) the damage severity is low, consisting of focal changes at the synaptic level such as hyperexcitability, increased membrane permeability (either through membrane disruption or receptor activity), and onset of excitotoxic conditions. During the acute phase (hours to several days), damage consists of further receptor alterations, activation of proteases and apoptotic pathways, mitochondrial dysfunction, and prolonged excitotoxic conditions. During the subacute phase (several days to weeks), regional deficits within the brain are observed due to cellular dysfunction, prolonged neuroinflammation, and onset of phosphorylated tau accumulation. During the chronic phase (months and beyond), major *behavioral* deficits and neuropsychiatric conditions are observed, including, but not limited to, impaired learning and memory, depression, and prolonged headaches. The neuropathology of chronic traumatic encephalopathy is also observed during this time (Guerriero et al., 2015; MacFarlane and Glenn, 2015). Created with BioRender.com.

The timeline of cellular responses that occur after brain impact spans four phases: hyperacute (minutes to hours), acute (hours to several days), subacute (several days to weeks) and chronic (months and beyond; Guerriero et al., 2015; MacFarlane and Glenn, 2015). During the hyperacute phase, affected neurons undergo substantial and abnormal electrical and cellular activity leading to maladaptive remodeling, potentially as an attempt to regain homeostasis following impact. While the affected cells work to re-establish internal homeostasis during each phase, if damage were to remain unresolved (ex. further damage, or severity of initial damage) the pathology continues and moves into the next phase. As each phase progresses to the next, the damage severity increases. This can ultimately lead to the major neurodegeneration observed in pathologies associated with head trauma (Figure 1).

### Hyperacute Changes to the Pre-synaptic Terminal

The promotion of excitotoxic conditions requires prolonged release and presence of glutamate within the synaptic cleft, leading to overactivation of *N*-Methyl-d-aspartate receptors (NMDAr) and of α-amino-3-hydroxy-5-methyl-4-isoxazolepropionic acid receptors (AMPAr) on the post-synaptic membrane. *In vitro* neuronal stretch models have demonstrated an increase in membrane permeability within minutes after stretch through dysregulated ionic channels or the membrane physically rupturing (Smith et al., 1999; Weber, 2012; Siedler et al., 2014). Wolf et al. (2001) found that within minutes following stretch strain, an increase in intracellular Ca^2+^ was observed with levels that continued to rise during the time course of the experiment (1-20 min). This increase was dependent on the influx of Na^+^ as blockade of the voltage gated Na^+^ channels with tetrodotoxin inhibited this rise in intracellular Ca^2+^ (Wolf et al., 2001).

**FIGURE 2:**
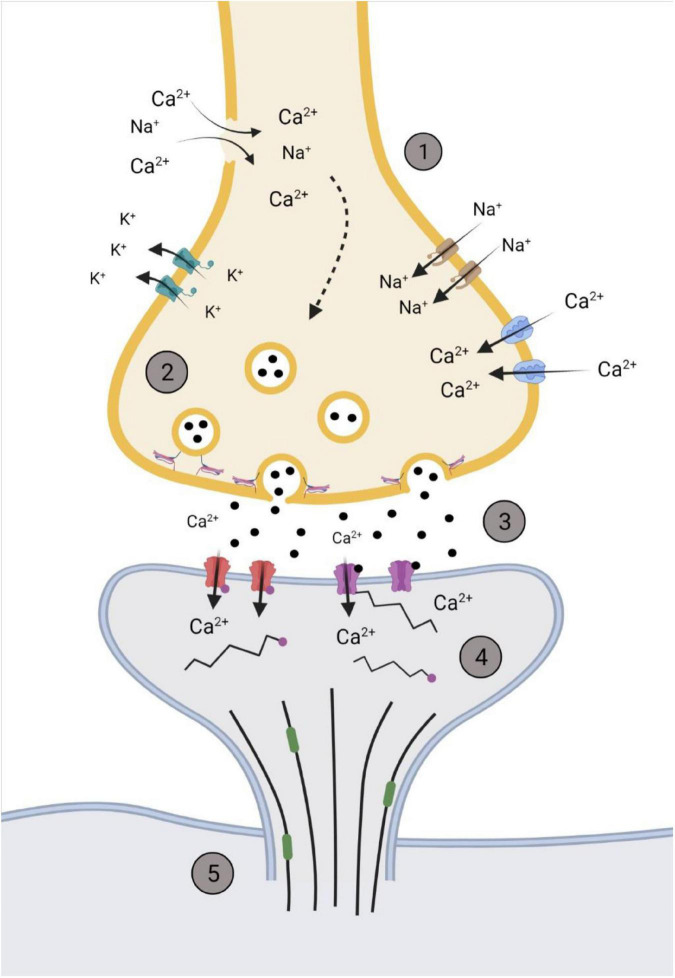
Representation of the synapse within the hyperacute phase after impact. (1) Membrane permeability increases soon after impact through the influx/efflux of ions via voltage gated channels or rupturing of the pre-synaptic membrane. (2) The increase in Ca^2+^ primes glutamate filled vesicles and increases the rate of fusion and release of glutamate. (3) Increased and sustained release of glutamate (black dots) into the synaptic cleft activates NMDAr NR2B subunits (purple) and increases post-synaptic concentrations of Ca^2+^. This activates various kinases that phosphorylates AMPA GluR1 subunits (red), further promoting the influx of Ca^2+^. (4) Post-synaptic cytoskeleton alterations occur through phosphorylation (pink dots), with disinhibition of *remodeling* proteins, such as end-binding protein 3 (green), occurring to allow for the restructuring of the dendritic spine cytoskeleton. (5) Downstream cascades from the influx of Ca^2+^alter microtubule dynamics within the cytoskeleton of the neuron. Created with BioRender.com.

Increased intracellular Ca^2+^ in the pre-synaptic neuron prime the readily releasable pool of synaptic vesicles and the SNARE complex that mediates the fusion of vesicles to the membrane wall via various docking proteins (Hackett and Ueda, 2015; Kaeser and Regehr, 2017; Chen et al., 2021). Carlson et al. (2016) found that 6 h after cortical impact to rodents, there was a noticeable increase in SNAP-25 complex, as well as syntaxin, that steadily decreased over a 2-week period. This is of importance as SNAP-25 and syntaxin are two of the three main SNARE proteins responsible for vesicle-membrane fusion at the pre-synaptic terminal (Kaeser and Regehr, 2017; Chen et al., 2021). Ahmed et al. (2012) found that within 30 min following stretch damage to *Drosophila* motor neurons, there were elevated levels of Synaptotagmin, a Ca^2+^ sensitive vesicle trafficking protein that interacts with SNAP-25, within the pre-synaptic terminal. These synaptic changes, along with changes to the post-synaptic neuron, are summarized in Figure 2.

### Hyperacute Inflammatory Response

Neuroinflammation plays a significant role in modulating the damage after brain injuries, with the activation of numerous proinflammatory cytokines such as interleukin-1, 6, tumor necrosis factor-α (TNF- α), and interferon-γ reported within the hyperacute phase following injury (Patterson and Holahan, 2012; Smith et al., 2012; Xiong et al., 2018). Recently, attention toward molecular recognition of damage has been gaining interest as possible mechanisms for signaling the immune response after injury. Toll-like receptors (TLR) are a class of pattern recognition receptors that activate and mediate the production of cytokines involved in proinflammatory response (Vaure and Liu, 2014). TLR-4, specifically, has been shown to influence neuronal excitability through cytokine-enhanced NMDAr currents in hippocampal cultures (Balosso et al., 2014). In the context of TBI, however, TLR-4 enhances neuronal excitability through upregulation of Ca^2+^-permeable GluR1, and not NMDAr, hours after impact (Li et al., 2015; Korgaonkar et al., 2020). Using the fluid percussion injury (FPI) model in juvenile rats, Li et al. (2015) demonstrated elevated neuronal expression of TLR-4 within the hippocampus 4 h after impact, with a peak at 24 h, then returning to similar levels as 4 h at 3 days post-impact. This early increase in TLR-4 expression was accompanied with enhanced non-NMDA excitatory postsynaptic currents in granule cell and mossy cells at 3 days. The contribution of TLR-4 to increased neuronal excitability is through the downstream effector of nuclear factor kappa B (NF-κB; Vaure and Liu, 2014). In neurons, increased synaptic expression of NF-κB has been shown to promote excitatory synapses and spinogenesis through NMDAr regulated Ca^2+^ influx (Boersma et al., 2011). In glial cells, TLR-4-activated NF-κB has downstream effects on the upregulation on proinflammatory factors such as interferon-beta (IFN-β) and TNF-α (Vaure and Liu, 2014). Overproduction of these factors in microglia have been shown to promote excitotoxic conditions by downregulating excitatory amino acid transporter-2, thus reducing glutamate reuptake from astrocytes (Sitcheran et al., 2005). Another mechanism includes the TNF-α induced increase in AMPAr lacking GluR2, increasing the Ca^2+^ permeability (Stellwagen et al., 2005; for review see Olmos and Lladó, 2014).

### Maladaptive Role of Inhibition During Hyperacute Phase

While not directly in the scope of this review paper, the role of γ-aminobutyric acid (GABA) in the promotion of excitotoxic conditions is important. Immediately following FPI (i.e., within minutes of impact to tissue fixation), Toth et al. (1997) demonstrated that large basket-like cells within the granular layer of the rat hippocampus were damaged, as evident from positive Gallyas silver staining. The neighboring granule cells, however, showed no signs of damage indicating that, due to their size, these GABAergic interneurons were at more risk of damage compared to the tightly packed granule cells. Further, the authors note that the damage to GABAergic interneurons immediately after impact can further promote the state of hyperexcitability as there is limited inhibitory signals within this region of the hippocampus (Toth et al., 1997). More recently, studies have shown further downregulation of GABAergic subunits within the rat hippocampus, particularly α1, α2, γ2, and δ, 6 h following FPI (Raible et al., 2012; Drexel et al., 2015). Interestingly, both groups found upregulation of α4 subunits, with Raible et al. (2012) showing increase in the ipsilateral side, but not contralateral, and Drexel et al. (2015) showing increase in both ipsilateral and contralateral to region of impact. Considering how the α1, α2 and γ2 GABA subunits regulated phasic inhibition, and the α4 subunit regulates tonic inhibition, the increase in α4 subunit expression could be an indication of a compensatory mechanism to counteract the hyperexcited state occurring after impact (Drexel et al., 2015).

### Electrophysiological Changes Following Impact

Excitotoxic conditions arise from the prolonged release of glutamate into the synaptic cleft with subsequent binding to and activation of post-synaptic receptors. Having established an increase in membrane permeability, which leads to influx of Na^+^ and Ca^2+,^ as well as the efflux of K^+^ (MacFarlane and Glenn, 2015), one would assume that the dysregulation of ions across the membrane could be enough to trigger aberrant depolarizations and produce a hyperactive state shortly after trauma. However, much like the dynamics of the pre-synaptic terminal, there remains limited research into the hyperacute phase of TBI (Cohen et al., 2007). This could be in part due to the complexity of techniques from one study to another, or experimental limitations associated with electrophysiological recordings from slice preparations in such a protracted timeframe. Nonetheless, there have been a handful of studies looking at the electrophysiological properties that occur within the hyperacute phase following TBI.

Using microelectrode arrays to measure neural population activity following cortical compression, Ding et al. (2011) found a time-dependent shift in electrical activity after compression. Within minutes of injury, there was a depression in cortical activity, with a gradual rise in excitability within an hour post injury, eventually resulting in cortical hyperexcitability by the 2-h mark. Similarly, using an *in vitro* stretch model, it was shown that within 30 min there was a reduced occurrence of spontaneous action potentials in neurons that were damaged (Magou et al., 2015). Interestingly, the authors found that in the adjacent neurons that did not experience stretch damage, there was an increase in action potentials generated within that timeframe. This could potentially indicate that due to imbalance of ionic homeostasis immediately after trauma, the depression of electrical activity could act as a primer for the hyperexcitability experienced shortly after (Berger et al., 2008; Ding et al., 2011). This enhanced activity in adjacent neurons could propagate hyperexcitatory conditions, potentially initiating the secondary, maladaptive intracellular pathways associated with TBI.

The release of ions following injury has also been shown to be enhanced within 24 h after injury and alter electrophysiological properties. In a rather paradoxical way, D’Ambrosio et al. (1998) demonstrated a loss of long term potentiation (LTP), but not long term depression (LTD), in hippocampal slice recordings, 24 h after FPI in rats. They found that rats who received FPI had higher thresholds required to evoke population spikes within the CA1. However, previous work from Katayama et al. (1990) demonstrated, *in vivo*, a massive increase in extracellular K^+^ following FPI resulting in neuronal hyperexcitability. D’Ambrosio et al. (1998) attribute their opposite finding to differences in the microenvironments between *in vivo* and *in vitro* settings. They note that the elevated efflux of K^+^ abolishes LTP as the sudden overactivation of NMDAr and firing of the post-synaptic neuron leaves the neuron unable to undergo further potentiation after injury; something not reproducible in an *in vitro* setting (D’Ambrosio et al., 1998). Interestingly, a year later D’Ambrosio et al. (1999) demonstrated altered neuronal activity due to excessive accumulation of extracellular K^+^ 24 h after FPI in hippocampal slices. This accumulation led to a hyperexcited state in CA3 neurons, as evident by the higher percentage of burst discharges in the post-FPI slices. The authors also note a significant loss of inward rectifying K^+^ currents from recruited glia cells. These findings indicate a disruption to mechanisms responsible for maintaining extracellular homeostasis, such as the decreased K^+^ clearance from glia cells plus the prolonged release of K^+^ from hyperactive neurons (D’Ambrosio et al., 1999).

A report by Ross and Soltesz (2000) examined the electrophysiological properties of interneurons in the dentate gyrus after lateral FPI. Within the dentate gyrus, there was an observed increase of spontaneous interneuron firing rates due to Na^+^/K^+^-ATPase-related depolarizations. This increase in interneuron firing rates after impact led to increased frequency and amplitude of spontaneous inhibitory postsynaptic potentials in dentate gyrus granule cells. It is important to note that this increase in spontaneous inhibitory postsynaptic potentials occurs simultaneously to a decrease in miniature inhibitory postsynaptic current frequency. The authors describe this post-FPI increase in interneuron depolarization and increased GABAergic transmission could increase the efficacy of incoming excitatory signals to further promote interneuron firing after impact. This rather paradoxical mechanism has been reported in previous models of hyperexcitability, such as kindling seizures, chronic stress-induced seizures, and febrile seizures (Nusser et al., 1998; Chen et al., 1999; MacKenzie and Maguire, 2015).

### Hyperacute Changes to the Post-synaptic Terminal

Using animal models of TBI, it has been shown that extracellular glutamate levels rise drastically within minutes after impact and remained elevated for hours (Faden et al., 1989; Folkersma et al., 2011; Xiong et al., 2019). Along the post-synaptic membrane, AMPAr and NMDAr become over-activated from the increased presence of glutamate within the synaptic cleft. The GluR1 subunit of AMPAr plays a crucial role in LTP and membrane conductance under normal conditions; however, they have been implicated in progressing neurodegenerative diseases under pathological conditions (Spaethling et al., 2012). Unlike the GluR2 subunit, which limits membrane permeability of Ca^2+^, phosphorylated GluR1, via Ca^2+^/calmodulin-dependent protein kinase II (CaMKII) and PKC, increases membrane permeability to Ca^2+^ and promotes the upregulation of GluR1 insertion into the membrane and the influx of Ca^2+^ (Isaac et al., 2007). In the context of TBI, *in vitro* stretch models reveal an increase in GluR1 phosphorylation within hours of injury and increased levels of intracellular Ca^2+^ (Spaethling et al., 2008, 2012). Phosphorylation of GluR1 is mediated through NMDAr activity, specifically the NR2B subunit activity, as blocking the NR2B subunit blocked the rise in Ca^2+^ levels and reduced levels of phosphorylated GluR1 in stretched neurons (Spaethling et al., 2012). This has been observed in animal models of TBI, with increased CaMKII autophosphorylation and GluR1 observed within the hippocampus as early as 15 min after using weighted drop controlled cortical impact (CCI; Schumann et al., 2008) and 1 h post FPI (Atkins et al., 2006).

Calcium influx via the activation of NMDAr is crucial for synaptic plasticity and activating various biochemical pathways responsible for neuronal physiology. However, sustained activation of NMDAr promotes pathological secondary cascades which underlie the onset of neurodegenerative diseases (Kalia et al., 2008; Shohami and Biegon, 2014). Within the hyperacute phase of TBI, phosphorylated NR2B levels begin to rise and promote the upregulation of other NMDAr subunits, such as NR1 and NR2A (Schumann et al., 2008). The NR2B subunit is responsible for high influxes on Ca^2+^ into the neuron, with dysregulation of this subunit leading to excitotoxic conditions (Arnsten et al., 2021). In cultured neurons, the loss of functional connectivity is primarily mediated through activation of NR2B subunit (Patel et al., 2014). The NR2B subunit appears to be the focal subunit of potential neurotoxic effects, as administration of inhibiting kinases involved in regulating the receptor reduced the elevated levels of phosphorylated NR2B and improved long term neurological function after trauma (Schumann et al., 2008; Zhou and Sheng, 2013; Patel et al., 2014).

## Dendritic Spine Classification

Dendritic spines are the primary site for excitatory synaptic communication between two neurons and their morphology can greatly dictate neural function. Dendritic spines are dynamic in their morphology, constantly shifting between phenotypes depending on the surrounding environment or internal cellular conditions (Hering and Sheng, 2001). The constant remodeling and development of spines is termed spinogenesis with these changes taking place in the time frame of seconds to minutes to days to weeks (Calabrese et al., 2006). The remodeling and retracting of spines is a highly conserved trait across species, with evidence to show spinogenesis rates changing during learning and memory (Tønnesen and Nägerl, 2016; Luengo-Sanchez et al., 2018), disease progression (Calabrese et al., 2006; Aguayo et al., 2018; Jamjoom et al., 2021) and naturally with age (Benavides-Piccione et al., 2013).

**FIGURE 3:**
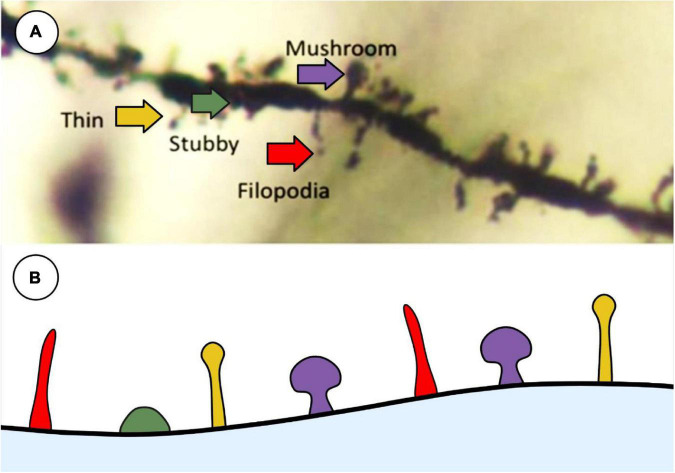
Representative image of main spine types that are commonly used in histological analysis described by Peters and Kaiserman-Abramof (1970). **(A)** Golgi-stained neuron showcasing the 4 main types of spines: thin (yellow), stubby (green), filopodia (red), and mushroom (purple). **(B)** Schematic representation of the types of spines to illustrate differences between shapes. Created with BioRender.com.

In their 1970 paper, Peters and Kaiserman-Abramof (1970) described the three main categories of spine type: thin, stubby and mushroom. Thin spines represent a transitory phenotype that are primed to develop into mushroom-type spines through repeated synaptic activity (Pchitskaya and Bezprozvanny, 2020). Stubby spines represent immature spines and are less likely to be a place of synaptic communication between neurons (Helm et al., 2021). Mushroom spines are considered to represent an excitatory synapse and are associated with synaptic plasticity achieved through LTP (Jaworski et al., 2009; Helm et al., 2021). Using modern neuronal reconstruction, Luengo-Sanchez et al. (2018) characterized the spine morphology in human cortical pyramidal neurons using three dimensional meshes on individual spines and quantitative features such as, but not limited to, height, growth direction of the spine, and volume. This group was able to categorize dendritic spines into six distinct clusters based on the Bayesian information criterion, which contained sub-sets of spine shapes based on the authors described criterion. Bokota et al. (2016) produced 10 distinct spine clusters using automatic three-dimensional dendritic spine reconstruction, with each cluster containing a range of spine morphology subsets. While modern imaging techniques show that spine classification exists on a continuum at any one time (Rodriguez et al., 2008; Bokota et al., 2016; Luengo-Sanchez et al., 2018), the categories set forth by Peters and Kaiserman-Abramof (1970) are still widely used to classify spines when performing histological analyses (Figure 3).

### Dendritic Spine Physiology

The cytoskeletal filaments within the spine head are made up of an intricate network of actin filaments that form the basis for morphological spine remodeling. These filaments, along with the molecular machinery involved in the dynamics of the spine cytoskeleton, act independently from the machinery in neighboring spines (Newpher and Ehlers, 2009). The ultrastructural changes within a single dendritic spine involve the recruitment of several protein kinase families that regulate actin filament dynamics within the spine head (Meng et al., 2003; Jaworski et al., 2009; Aguayo et al., 2018; Reza-Zaldivar et al., 2020). Lim-kinase (LIMK), for example, is a major family of protein kinases that regulate the shape of dendritic spines and their function and plays a major role in LTP (Meng et al., 2003). Through the influx of Ca2^2+^ via NMDAr, CaMKI, and CaMKII stimulate the activity of Rac1. The stimulation of Rac1 has downstream effects of promoting LIMK-1 activity which ultimately leads to actin polymerization and the maintenance of spine morphology [for full review see Saneyoshi et al. (2010)]. The rate in which LIMK-1 is activated and suppressed is achieved by brain derived neurotrophic factor (BDNF) and microRNA-134 (miRNA-134), respectfully, however the exact mechanisms in which this operates is not well understood (Schratt et al., 2006; Saneyoshi et al., 2010; Reza-Zaldivar et al., 2020).

In addition to the regulation of dendritic spine actin, the activity along the synaptic membrane and the scaffolding proteins that make up the post-synaptic density (PSD) regulate cytoskeletal dynamics of the spine. The PSD is roughly 50 nm wide and is enriched in a wide variety of proteins responsible for synaptic integrity and regulating what enters the neuron (Harris and Kater, 1994). One of the most important synaptic proteins responsible for synaptic stability is PSD-95. PSD-95 is a scaffolding protein responsible for the regulation of AMPAr and NMDAr, as well as neuroligins and neurexins responsible for anchoring the pre-synaptic and post-synaptic terminals together (Keith and El-Husseini, 2008; Jeong et al., 2019). Knockdown models of PSD-95 have shown decreased spine numbers, altered formation of functional synapses and decreased rates of spinogenesis (Jeong et al., 2019). The overexpression of PSD-95 promotes development of excitatory synapses along the dendrite (Levinsoni et al., 2005).

### Dendritic Spine Receptors

The basis for synaptic plasticity and LTP involves the movement of ions across the membrane through AMPAr channels and NMDAr channels. NMDAr have been largely implicated in the consequences of excitotoxicity, however there is mounting evidence to suggest the upregulation of the AMPA GluR1 subunit contributes to excitotoxic conditions (for review see Guerriero et al., 2015). AMPAr are the primary fast excitatory receptor within the nervous system, consisting of 4 subunits, GluR1 to GluR4, which form tetrameric complexes that have distinct receptor binding subtypes. Most AMPAr contain the Ca^2+^-impermeable GluR2 subunit, however a subset of AMPAr contain the Ca^2+^-permeable GluR1 subunit (Song and Huganir, 2002). Once glutamate is bound, the pore of the receptor opens allowing for cations (Na^+^ and Ca^2+^) to move across the membrane resulting in depolarization (Chater and Goda, 2014). NMDAr consist of three main subfamilies, GluN1, N2, N3, with multiple subunits within each family. A combination of subfamilies makes up the NMDAr channel, with GluN1 and N2 subunits pairing together, or GluN2 and N3 together. Acting as a coincidence detector, the opening of NMDAr channels requires the depolarization of the neuron as well as the binding of glutamate followed by the removal of the Mg^2+^ ion within the membrane-spanning domain of the channel (Paoletti et al., 2013). Once Mg^2+^ is removed, the NMDAr becomes permeable to Na^+^ and Ca^2+^. NMDAr activation and Ca^2+^ play a crucial role in the LIMK pathway through activating CaMKII and remodeling the actin cytoskeleton of the dendritic spine (Newpher and Ehlers, 2009; Saneyoshi et al., 2010). The influx of Ca^2+^ into the neuron is required for normal cellular functioning through the activation of various enzymes, protein complexes, and immediate early genes (Paoletti et al., 2013). Too large of an influx of Ca^2+^, however, has been established as a main factor in the onset of excitotoxic conditions that put the neuron at risk of cell death, and ultimately the onset of various neurodegenerative diseases.

### Cytoskeleton Changes Within the Dendritic Spine After Impact

**FIGURE 4:**
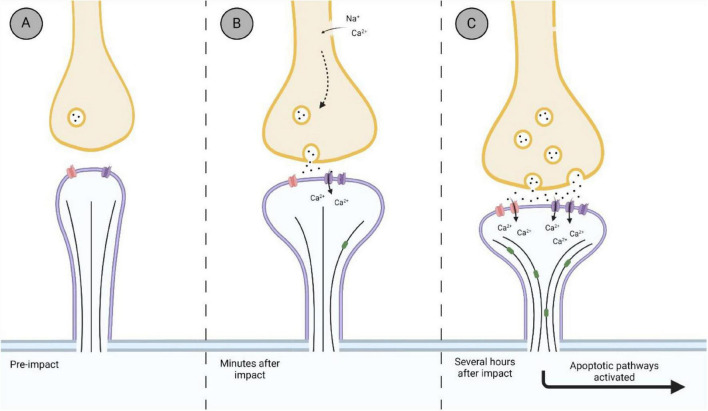
Representation of the biological timeline of the synapse after impact. **(A)** Thin synapse before an impact. Thin spines are considered a transitory spine phenotype, with the potential to transition in an excitatory synapse. **(B)** Within minutes after impact, synaptic changes within the hyperacute phase after impact. Membrane ruptures and ionic imbalances promote the hyperexcitability of the synapse, with an influx of Ca^2+^ into the post-synaptic terminal via NMDAr (purple). **(C)** Further damage to the synapse due to hyperexcitability and sustained release of glutamate from the pre-synaptic terminal is observed several hours after impact. Increased influx of Ca^2+^ into the post-synaptic terminal promotes the further upregulation of NMDAr, as well as upregulating GluR1 subunit of AMPAr (red). Disinhibition of synaptic reconstruction proteins, such as EB3 (green), occurs as restricting proteins become inactivated, promoting excitatory mushroom spine phenotype. Hyperexcitability promotes neurodegenerative pathways via overworked mitochondria and damage to the microtubule cytoskeleton of the neuron. Created with BioRender.com.

Within the post-synaptic membrane, the synaptic architecture is stabilized primarily through PSD-95 which is regulated through the activity of NR2B subunits (Szydlowska and Tymianski, 2010; Frank et al., 2016; Jeong et al., 2019). PSD-95 has also been shown to restrict the remodeling of dendrites by mediating the activity of end-binding protein 3 (EB3); a protein responsible for growth and microtubule dynamics (Patel et al., 2019). EB3 is a well conserved member of the EB family responsible for restructuring dendritic spine cytoskeleton, maintenance of excitatory synapses, shuttling organelles into dendritic spines, and has shown to be involved in LTP (Jaworski et al., 2009; Penzes et al., 2009). The activity of PSD-95 is regulated by phosphorylation on cyclin-dependent kinase 5 (cdk5; Keith and El-Husseini, 2008). Park et al. (2013) demonstrated an increase in phosphorylated PSD-93, a similar scaffolding protein to PSD-95, several hours following FPI in the rodent cortex. These proteins are considered deactivated once phosphorylated (Nowacka et al., 2020). This could suggest that following TBI, the disruption of PSD-95/93 allows for the remodeling of the dendrite and spine through proteins such as EB3 (Figure 4), as their activity is no longer inhibited (Patel et al., 2019). Very few studies have investigated the remodeling of dendritic spine morphology post-TBI within the hyperacute phase. Pijet et al. (2019) found that 24 h after CCI, dendritic spines in the ipsilateral cortex became shorter and wider, a characteristic of excitatory mushroom-type spines (Jontes and Smith, 2000). The expression of shorter-wider spines was accompanied by an increase in the matrix metalloproteinase-9 (MMP-9). This extracellular protease has a role in synaptic plasticity as it is present in excitatory synapses within the hippocampus, cerebellum and cerebral cortex (for review see Michaluk and Kaczmarek, 2007). Moreover, inhibition of MMP-9 activity has been shown to make spines resistant to excitotoxic damage from TBI as well as post-impact-induced seizures (Michaluk and Kaczmarek, 2007; Hayashi et al., 2009; Pijet et al., 2019). Expression of MMP-9 has been seen to increase 30 min after TBI, and remaining elevated for hours and days (Wang et al., 2000; Truettner et al., 2005; Hayashi et al., 2009; Pijet et al., 2018). While not reported in Pijet et al. (2018), it would be interesting to see if the increase in MMP-9 expression 30 min after CCI would be accompanied by a change in dendritic spine phenotype, similar to what they found a year later at 24 h after impact.

## Damage to the Dendrite Outside of the Spine Head Following Impact

The large influx of Ca^2+^ that follows TBI activates various downstream pathophysiological cascades that put the neuron at risk of degeneration. One of these pathways includes alterations to the neuronal microtubule network. Microtubules provide both cytoskeletal structuring to the neuron, as well as act as a “molecular highway” for proteins and organelles to travel throughout the neuron. Calpain is a family of calcium-activated proteases involved in numerous physiological functions ranging from modifications to receptors and channels, activation of apoptotic proteins and regulation of structural proteins (Saatman et al., 2010). The activation of calpain can occur within minutes of injury and be sustained for several days (Saatman et al., 2010). In particular, calpain-2 has been shown to have neurodegenerative consequences several hours post-TBI, as evidence by increased calpain-2 levels correlating with the activation of apoptotic pathways in mice (Wang et al., 2018). Calpain-2 also disrupts microtubule dynamics by phosphorylating tau, the accumulation and aggregation of which has been well documented in the onset of tauopathy’s such as chronic traumatic encephalopathy and Alzheimer’s disease (Goldstein et al., 2012; Baudry and Bi, 2016; for review see McKee et al., 2013).

Along with activation of neurodegenerative pathways such as calpain-2, the influx of Ca^2+^ that follows a TBI places stress upon the mitochondria of the neuron (Watts et al., 2015). In data not shown, Griesemer and Mautes (2007) noted an increase in adenosine triphosphate levels 1 h post closed head impact, with a decrease occurring by hour 4. Overworked mitochondria fail to buffer the rise in intracellular Ca^2+^ levels and as a result damaging reactive oxygen species, such as H_2_O_2_, are created (Vercesi et al., 2018). While these secondary pathophysiological pathways take time to develop, the initial onset of the sequelae begins with a moment of disruption shortly after impact. Further investigations into the hyperacute neuronal responses to TBI will be beneficial in the development of possible therapeutic targets and better understanding of how these chronic diseases initiate.

## Conclusion

The hyperacute phase that follows TBI has been relatively understudied when it comes to understanding the dynamics of head trauma. The changes at the level of the synapse showcase the disrupted communication between neurons and how it reacts to the change in the environment. These subtle changes at the synapse amplify as further damage occurs, progressing the pathological pathway that is seen in the sub-acute and chronic phases of diseases associated with TBI. Understanding where the biochemical changes begin following head trauma could direct the field of research to the development of effective treatments and therapeutics soon after an impact has occurred.

## Author Contributions

BH contributed to the research and drafting of manuscript. MRH contributed to the revisions and final approval of manuscript. Both authors contributed to the article and approved the submitted version.

## Conflict of Interest

The authors declare that the research was conducted in the absence of any commercial or financial relationships that could be construed as a potential conflict of interest.

## Publisher’s Note

All claims expressed in this article are solely those of the authors and do not necessarily represent those of their affiliated organizations, or those of the publisher, the editors and the reviewers. Any product that may be evaluated in this article, or claim that may be made by its manufacturer, is not guaranteed or endorsed by the publisher.
